# Supplementary material for: Structure-Based Pharmacophore Modeling, Virtual Screening, Molecular Docking and Biological Evaluation for Identification of Potential Poly (ADP-Ribose) Polymerase-1 (PARP-1) Inhibitors
Source: Molecules. 2019 Nov 22;24(23):4258. doi: 10.3390/molecules24234258 (PMC6930522; doi:10.3390/molecules24234258)
Supplement: Supplementary file 1 [file molecules-24-04258-s001.pdf]

Supplementary Materials for

Structure-Based Pharmacophore Modeling, Virtual Screening, Molecular Docking and Biological Evaluation for Identification of Potential Poly(ADP-ribose)polymerase-1 (PARP-1) Inhibitors

Yunjiang Zhou, Shi Tang, Tingting Chen and Miao-Miao Niu*

Department of Pharmaceutical Analysis, State Key Laboratory of Natural Medicines, School of Basic Medicine and Clinical Pharmacy, China Pharmaceutical University, Nanjing 210009, China
Tel.: +86 25 83271080; fax: +86 25 83271046
*Correspondence: niumm@cpu.edu.cn; Tel.: +86 25 83271080

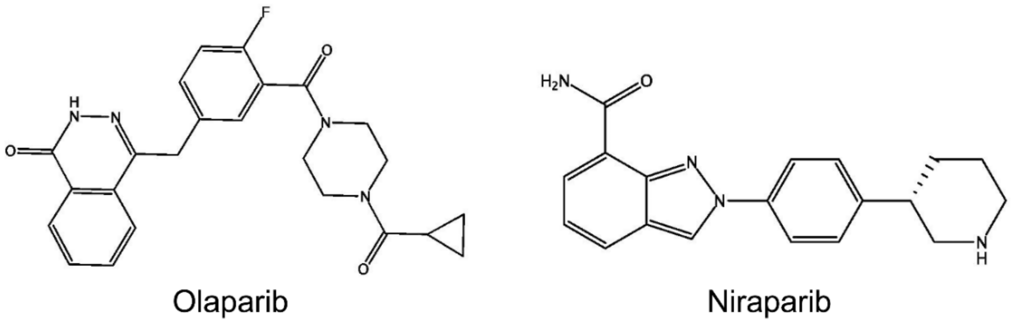

Figure S1. Structures of the PARP-1 inhibitors olaparib and niraparib.

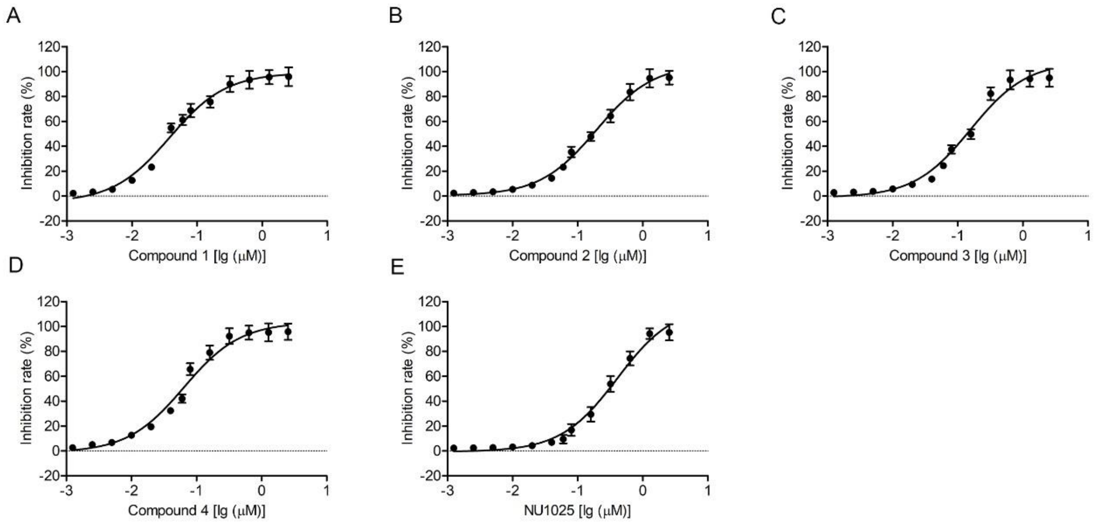

Figure S2. PARP-1 inhibition of compounds 1-4 and NU1025 measured by the PARP-1 enzyme assay.

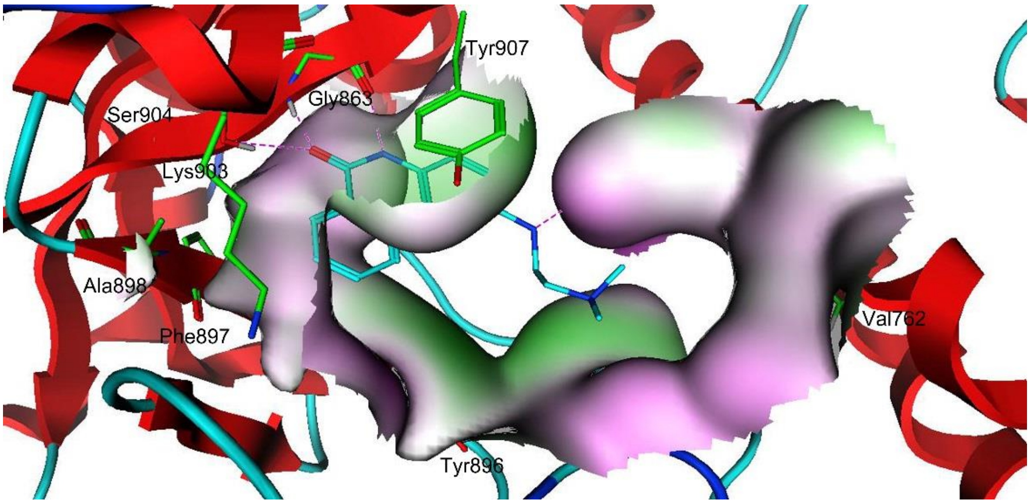

Figure S3. The three-dimensional (3D) ligand-protein interaction diagram for the binding site of PARP-1 with compound 2. The active site residues are shown in green stick form. The hydrogen-bond network with protein residues is represented in red dotted lines. Compound 2 is color-coded by cyan.

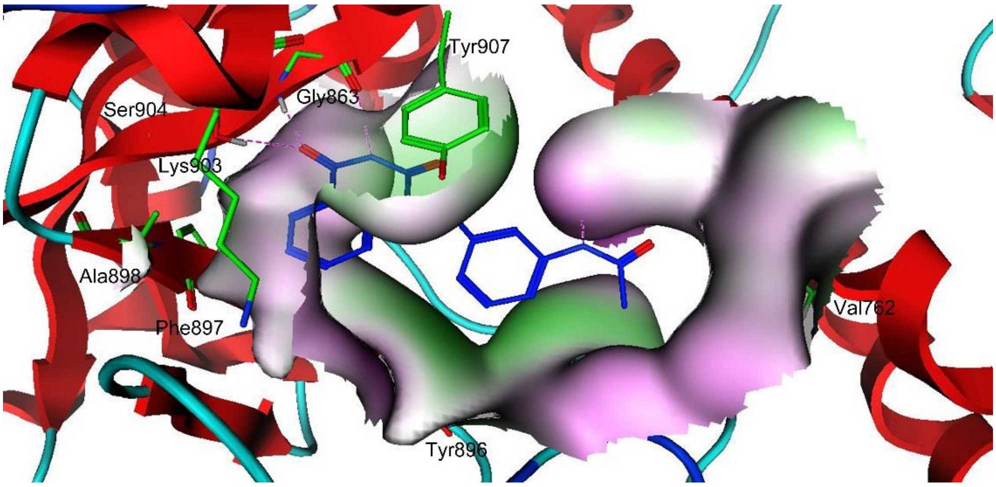

Figure S4. The three-dimensional (3D) ligand-protein interaction diagram for the binding site of PARP-1 with compound 3. The active site residues are shown in green stick form. The hydrogen-bond network with protein residues is represented in red dotted lines. Compound 3 is color-coded by blue.

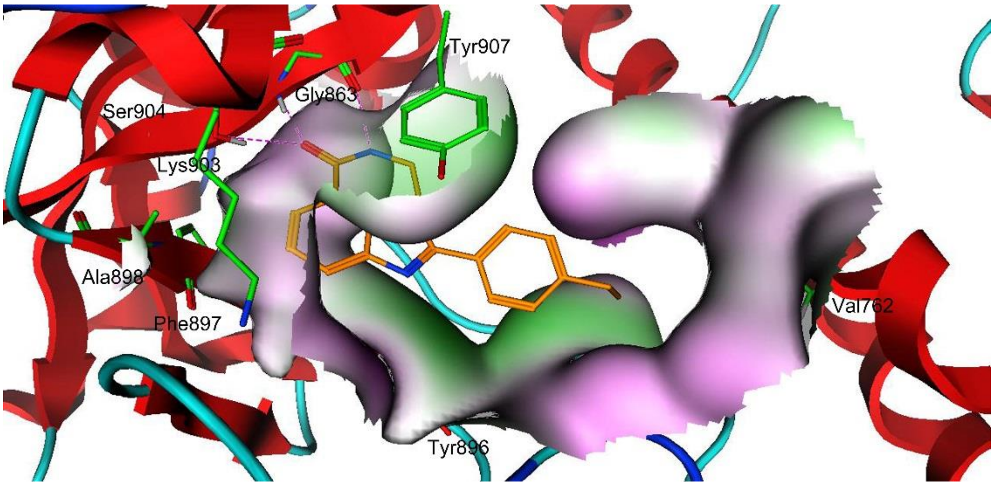

Figure S5. The three-dimensional (3D) ligand-protein interaction diagram for the binding site of PARP-1 with compound 4. The active site residues are shown in green stick form. The hydrogen-bond network with protein residues is represented in red dotted lines. Compound 4 is color-coded by orange.
